# Endocardium in Hypoplastic Left Heart Syndrome: Implications from In Vitro Study

**DOI:** 10.3390/jcdd9120442

**Published:** 2022-12-08

**Authors:** Zhiyun Yu, Ziyi Liu, Vidhya Ravichandran, Bonny Lami, Mingxia Gu

**Affiliations:** 1 Perinatal Institute, Division of Pulmonary Biology, Cincinnati Children’s Hospital Medical Center, Cincinnati, OH 45229, USA; 2 Center for Stem Cell and Organoid Medicine, CuSTOM, Division of Developmental Biology, Cincinnati Children’s Hospital Medical Center, Cincinnati, OH 45229, USA; 3 College of Medicine, University of Cincinnati, Cincinnati, OH 45267, USA

**Keywords:** hypoplastic left heart syndrome, endocardium, in vitro study, induced pluripotent stem cell (iPSC)

## Abstract

Endocardium lines the inner layer of the heart ventricle and serves as the source of valve endothelial cells and interstitial cells. Previously, endocardium-associated abnormalities in hypoplastic left heart syndrome (HLHS) have been reported, including endocardial fibroelastosis (EFE) and mitral and aortic valve malformation. However, few mechanistic studies have investigated the molecular pathological changes in endocardial cells. Recently, the emergence of a powerful in vitro system—induced pluripotent stem cells (iPSCs)—was applied to study various genetic diseases, including HLHS. This review summarized current in vitro studies in understanding the endocardial pathology in HLHS, emphasizing new findings of the cellular phenotypes and underlying molecular mechanisms. Lastly, a future perspective is provided regarding the better recapitulation of endocardial phenotypes in a dish.

## 1. Introduction

Hypoplastic left heart syndrome (HLHS) is a form of congenital heart disease characterized by a hypoplastic left-heart ventricle, mitral and aortic atresia/stenosis, and hypoplasia of the ascending aorta or arch [1]. Three main subgroups of the left ventricular phenotype were identified—a small number of cases were classified with a miniature left ventricle with anatomically normal valve structures. The “slit-like ventricle” subtype accounts for 24% of the cases, characterized by a flattened left ventricle with a thin left ventricle wall. All the hearts within this subtype had aortic atresia and mitral atresia. Another 70% of the cases present as a “small left ventricle cavity with the thickened parietal wall”. Most cases exhibited mitral stenosis/aortic stenosis (MS/AS) or mitral stenosis/aortic atresia (MA/AA). In addition to valve abnormalities, endocardial fibroelastosis (EFE) was frequently found, characterized as a thickening within the muscular lining of the chambers due to an increase in supporting connective tissue [2]. The endocardium lines the atrial and membranous ventricular septa and serves as the source of the mesenchymal cells that give rise to the atrioventricular valves [3]. During the early developmental stage, the endocardial–myocardial crosstalk was found critical for myocardial differentiation and trabeculation [4]. Notably, the high incidence of valve abnormality and EFE in HLHS cannot be solely explained by intrinsic myocardial defects, which underscored the possible fundamental role of the endocardial defect in the disease etiology.

## 2. Pathology of Endocardium in HLHS

The endocardium is made of specialized endothelial cells (ECs) located in the innermost layer of the heart wall. Endocardial cells are pivotal in heart development and congenital heart disease pathogenesis [5]. By undergoing the process termed endothelial-to-mesenchymal transition (Endo-MT), the endocardial ECs can be transformed to form the endocardial mesenchymal cushion, which gives rise to the ventricular septum and atrioventricular valves [6,7]. Previously, by combining analyses of human EFE tissue from HLHS patients and a rodent EFE model of heterotopic transplantation, it was demonstrated that the fibrogenic cells within EFE tissue originated from endocardial cells via aberrant Endo-MT, which was due to dysregulated bone morphogenetic protein (BMP) signaling [8]. Additionally, recent clinical observation reported an association between EFE formation and flow disturbance [9]. Endocardial ECs serve as an information hub sensing flow patterns and mechanical stress. The role of flow-induced Endo-MT in EFE pathogenesis was further validated using in vivo surgical models and in vitro EC culture under static or laminar flow patterns [10].

Moreover, signaling pathways within endocardial–myocardial interaction serve as important mediators for the trabeculation and maturation of cardiomyocytes. Single-cell analysis of a mouse model of ventricular non-compaction revealed the pathological role of the endocardium in cardiomyocyte proliferation and maturation by the dysregulation of angiogenic factors [11]. Recently, a single-cell transcriptome analysis of a human fetal heart elucidated that the majority of the genes harboring HLHS de novo mutation (DNM) genes were highly expressed in the endocardial cell population [12]. This finding, for the first time, brought people’s attention to the intrinsic defect of the endocardium that contributes to the disease etiology. All the evidence points to the central role of the endocardium in the critical cardiac development process (valve formation, cardiomyocyte proliferation) that is highly related to HLHS pathogenesis.

## 3. In Vitro Study of HLHS–iPSC Is an Emerging Technology in Disease Modeling

Genetic analysis on HLHS cohorts with multiple tools, including next-generation sequencing, has linked HLHS with complex genetic disorders [13,14]. A few identified candidate genes have been validated through robust genetic or functional studies [15]. A previous mouse model was developed with mutations in *Sap130* and *Pcdha9*; however, it was not sufficient to recapitulate the complex phenotypes of HLHS, and the penetrance was low [16]. Another surgically induced mouse model reinforced the importance of pathological hemodynamics in contributing to the disease progression, but it could not explain the heterogeneous genetic background of the disease [17]. Due to the strong but complicated genetic background of HLHS and the difficulty in obtaining human heart tissue for molecular and functional studies, the platform of induced pluripotent stem cells (iPSCs) provided a suitable alternative strategy due to its ability to carry genetic information from patients with HLHS. In 2006, Drs. Takahashi and Yamanaka found that differentiated somatic cells could be reprogrammed into an embryonic-like state via the forced expression of selected transcription factors [18]. iPSCs retain the complete genomic information from affected patients and are immortally proliferating. Over the decades, researchers have been able to visit disease-specific defects in various iPSC-derived cardiac cell types by utilizing efficient differentiation protocols. Till now, iPSC-derived cardiomyocytes have been successfully deployed for investigating HLHS disease mechanisms, including impaired differentiation, lower beating rate, disorganized sarcomere, and NOTCH1-dependent nitric oxide signaling deficiency [19,20,21,22,23]. However, except for various iPSC studies regarding general early cardiac progenitors and cardiomyocyte population, few in vitro studies have investigated the pathology of the endocardium in disease progression.

## 4. iPSC Modeling in Studying Endocardial Defects in HLHS

In a recent publication, Miao et al. extensively investigated the critical role of the endocardium in HLHS etiology [12]. Through single-cell RNA (scRNA) sequencing of a normal human fetal heart sample, they discovered that HLHS-associated DNMs were highly expressed in the endocardium, coronary and lymphatic ECs, which provided a robust rationale for targeting endocardium in HLHS pathogenesis. In their study, iPSC-derived endocardial endothelial cells (iEECs) purified by endocardial specific marker NPR3 were generated for the downstream in vitro study. Based on the differential expressed genes from scRNA transcriptome and in vitro functional assays, they demonstrated HLHS iEECs had several functional defects, including suppressed extracellular matrix (ECM) deposition, Endo-MT, VEGF signaling, and NOTCH activation. These functional pathways are vital players in valve formation and remodeling. 

**Figure 1:**
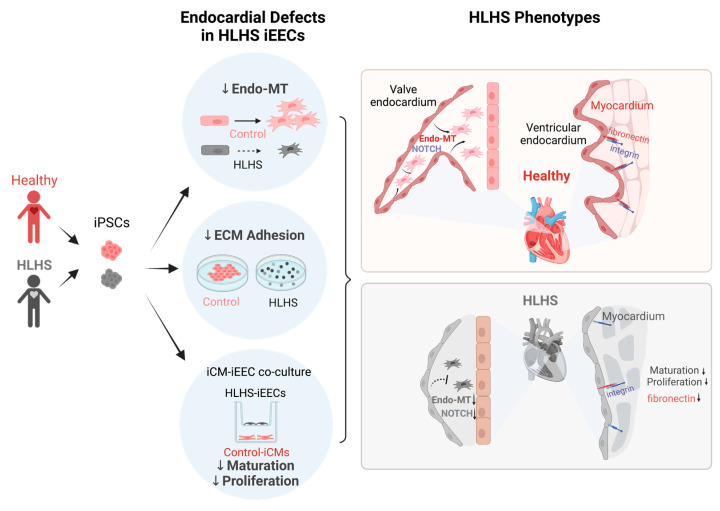
Using iPSC-derived endocardial EC (iEEC) to study the endocardial pathology in HLHS. iPSCs were initially derived from healthy donors and HLHS patients. iEECs were utilized to uncover various EC-related functional defects in HLHS-iEECs, including reduced Endo-MT, ECM adhesion, and the dysregulation of iPSC-derived cardiomyocytes (iCMs). The iEEC-based in vitro studies explained the disease phenotypes of HLHS, such as underdeveloped valve structure and unmatured and low proliferation of cardiomyocytes.

Furthermore, using an iEEC-cardiomyocyte co-culture system, they discovered that endocardial abnormalities could also cause reduced cardiomyocyte proliferation and maturation. Lastly, they uncovered the critical role of the gene FN1, coding for fibronectin (FN), in Endo-MT, cardiomyocyte proliferation, and maturation (Figure 1).

It is known that ECM deposition is critical for trabecular initiation and maturation. The endocardial ridges enriched in hyaluronic acid and FN can promote cardiomyocyte proliferation as the myocardial mass increases. At the later stage of trabeculation, the ECM is gradually attenuated when the endocardium reaches the outer myocardial layer, inducing a reduced proliferation of trabecular cardiomyocytes [24]. A previous study related to EFE in HLHS showed an increased deposition of ECM-rich fibrous tissue via Endo-MT [8]. However, it was also reported that the excessive fibroblasts in the EFE-like tissues were mainly derived from the epicardium [25]. Collectively, these studies indicated that the abnormal ECM deposition and Endo-MT in EECs contribute to the reduced proliferation and maturation of cardiomyocytes during the early phase of HLHS development. At the later stage, EFE may occur due to the participation of both endocardium and epicardium-derived fibroblasts and various environmental factors, including reduced blood flow and tissue hypoxia [26]. 

In summary, the combination of scRNA-seq and in vitro iPSC platform successfully unraveled the intrinsic defect in endocardium in HLHS at both cellular and molecular levels. The informative scientific findings also set a foundation for future regenerative strategies targeting endocardial function recovery.

## 5. Current Challenges of iPSC-EEC Modeling for HLHS

Over the years, transcriptomic profiling of ECs in different organs using single-cell technology revealed their organ-specificity [27,28,29]. Given the highly specialized property of the cardiac endocardium, the current iPSC-iEEC differentiation protocol may not generate mature endocardial cells that fully recapitulate their in vitro counterparts. Secondly, the potential of these iPSC-iEECs in giving rise to valve ECs (VECs) and valve interstitial cells (VICs) has yet to be determined. Therefore, there is a lack of investigation in understanding the HLHS-related phenotype of VICs and the VEC–VIC crosstalk in the field. Thirdly, a rapid de-differentiation of endocardial cells in vitro may lead to the loss of their cellular identities and make it difficult to maintain and passage in culture. Fourthly, the distinct characteristics of the RV and LV exist due to the different physiology and embryological origins [30]. However, the current iEEC model could not explain the chamber-specific pathology in HLHS. Thus, advanced iEEC differentiation strategies are needed to better recapitulate the endocardial defects in the disease. Lastly, only a limited number of HLHS iPSCs lines have been investigated, continued patient recruitment for additional iPSC samples is necessary to verify the current findings of HLHS endocardial pathology. In addition, the high-throughput multi-omics studies accompanied by patient tissue pathological examinations can also be utilized to validate the stem cell modeling system. Furthermore, it should be considered that the genetic cause was only identified in 20–30% of congenital heart disease cases. In addition to the known genetic factors contributing to the disease onset, the non-genetic environmental factors were thought to be critical in disease progression [31]. The non-genetic factors, including maternal pregestational or gestational diabetes, were found to be positively associated with the occurrence of HLHS [32,33,34]. Thus, the genetic and environmental etiology could be incorporated into a more complicated disease modeling using iPSCs. 

## 6. Future Perspective

Recently, several finely optimized protocols were published for differentiating iPSC into valvular cells characterized by conventional EEC markers (e.g., NPR3, CDH11, NFATC1, TGF-b2) and functional assays [35,36,37,38]. These iPSC-based strategies will benefit future investigations of EEC differentiation and VEC/VIC functionalities in the context of diseases with an intrinsic defect in the endocardium, such as HLHS. To differentiate valvular cells from iPSCs, Neri et al. showed an efficient induction of Mesp1+ cardiovascular progenitors via Wnt3a treatment, followed by cell sorting and further maturation on mouse embryonic fibroblasts. They also demonstrated the importance of FGF8 in triggering mesodermal cell fate toward endocardial cells and the Wnt activation for inducing Endo-MT and flow sensing in endocardial progenitors. The collagen gel culture assay provided additional insight into evaluating the Endo-MT capability of iPSC-derived VECs in a 3D structure [38]. Chang et al. reported their method of quick induction of primitive streak early mesodermal cells by BMP4 and Wnt3a treatment. They confirmed the essential role of BMP4 and TGF-β1 together with VEGF in inducing endocardial cushion cells and assessed the functionality of iPSC-VECs in fluid flow response [37]. Mikryukov et al. developed a VEC differentiation protocol that indicated the importance of BMP10 signaling in generating a cell population expressing the collection of markers that define the endocardial lineage. They further demonstrated that these iPSC-VECs acquired the capacity to induce a trabecular fate following co-culture with cardiomyocytes. At the transcriptomic level, a subpopulation of these iPSC-VECs showed a similar profile as primary human fetal endocardium based on scRNA sequencing analysis [35]. Additionally, Bao et al. reported for the first time that the iPSC-derived epicardial progenitors could differentiate into endocardial-like cells, indicating another clue to investigate the developmental origin of endocardial cells [36]. 

In addition to the iPSC-EEC differentiation in 2D, a more complicated cardiac structure comprising various cardiac lineages (e.g., myocardium, endothelium, epicardium) has been developed to study cell–cell communication during organogenesis. Over the years, cardiac organoids (COs) have emerged as a practical approach for studying sophisticated processes during normal cardiac development and disease. Several pathological conditions have been recapitulated by COs, such as myocardial infarction and cardiac hypertrophy, which are mostly related to myocardial defects [39]. Recently, Y. R. Lewis-Israeli et al. showed an optimized CO differentiation protocol that gave rise to multiple cardiac lineages with a heart field specificity [40]. Furthermore, the adjacent staining of EEC marker NFATC1 and myocardium marker TNNT2 in the developed COs showed a similar anatomic pattern to that in vivo, providing the possibility of further study of the endocardium–myocardium crosstalk. More delicate COs protocols are expected to include a more mature endocardial population within COs by considering the EEC-induction-related molecular factors mentioned above.

## 7. Conclusions

Over the decades, most studies about the pathological changes in HLHS endocardium have been focused on clinical anatomy and histological examination with limited implications regarding the cell type-specific defects and the underlying molecular mechanism. Due to the limited animal model that can recapitulate the complex phenotypes of this disease affecting multiple heart structures, iPSC-based in vitro studies provide a promising platform for investigating the intrinsic endocardial defects. Several abnormalities in HLHS iPSC-endocardial cells were identified—including compromised Endo-MT capacity, reduced ECM deposition, and the suppression of cardiomyocyte proliferation and maturation—providing a unique perspective of disease progression. Human iPSC-based protocols for endocardial cells and cardiac organoid differentiation are expected to be applied to the in vitro studies of specific endocardial pathobiology in HLHS.

## Data Availability

Not applicable.
